# Identification of the estrogen receptor GPER in neoplastic and non-neoplastic human testes

**DOI:** 10.1186/1477-7827-9-135

**Published:** 2011-10-05

**Authors:** Vittoria Rago, Francesco Romeo, Francesca Giordano, Marcello Maggiolini, Amalia Carpino

**Affiliations:** 1 Department of Cell Biology, Faculty of Pharmacy, University of Calabria, Italy; 2 Pathologic Anatomy Unit, Annunziata Hospital, Cosenza, Italy; 3 Department of Pharmaco-Biology, Faculty of Pharmacy, University of Calabria, Italy

## Abstract

**Background:**

Estrogen signaling is mediated by estrogen receptor beta isoforms in normal and neoplastic human testes. Recently, a G-protein-coupled-receptor (GPER) has been suggested as being involved in rapid responses to estrogens in different normal and tumor cells.

**Methods:**

This study investigated the GPER expression in paraffin-embedded samples from non neoplastic and neoplastic human testes (sex-cord stromal and germ cell tumors) by immunohistochemical and Western Blot analyses.

**Results:**

In control testes, a positive GPER immunoreactivity was detected in Leydig and in Sertoli cells while all germ cells were immunonegative. Furthermore, neoplastic cells of the Sertoli cell tumor, Leydig cell tumor, seminoma and embryonal carcinoma samples were all immunopositive. The immunoblots of testis extracts confirmed the results.

**Conclusions:**

These findings suggest that GPER could mediate estrogen signaling in both normal and transformed somatic cells of human testis, but they reveal a differential expression of the novel estrogen receptor in non neoplastic and neoplastic germ cells.

## Background

Estrogens exert their pleiotropic and tissue-specific effects on target cells through the differential expression of the classical estrogen receptors (ERs), ERα and ERβ, which mediate both genomic and rapid signaling events [1,2]. In addition, estrogens induce rapid non-genomic responses from membrane-associated receptors such as growth factor receptors and G protein-coupled receptors [3]. In the last years, a member of the 7-transmembrane G protein-coupled receptor family, GPR30, has been identified as a candidate to promote estrogen action in target cells [4-6] and different investigations have reported the expression of this novel estrogen receptor in a large variety of cell types [7]. Furthermore, GPR30 has been shown to have estrogen-binding affinity and to mediate estrogen-signal transduction events like calcium mobilization, kinase activation [5] and rapid transcriptional activation of early genes [8]. Therefore, despite some controversies regarding its biological role [9], GPR30 is now widely recognized as an estrogen receptor, with the new acronym G-protein coupled estrogen receptor GPER, by the International Union of Basic and Clinical Pharmacology [10]

In the human, GPER (GPR30) has been detected n heart, lung, liver, intestine, ovary, prostate, kidney, brain [11]. In addition, GPER (GPR30)has been also evidenced in neoplastic tissues from breast, endometrial and ovary cancers [12-14] as well as in breast [15-17], endometrial [18,19] ovarian [20] and thyroid carcinoma cells lines [21]. However, the association of the novel estrogen receptor with human cancer has only recently begun to be defined.

A large body of data indicated that estrogens regulate testis physiology [1] and they are also involved in male gonadic diseases, including cancer [22,23]. However, GPER signaling in human testes is still scarcely known. Therefore, the aim of this study was to investigate the pattern of GPER cellular distribution in non neoplastic and neoplastic human testes.

## Methods

### Patients

The investigation was performed on formalin-fixed and paraffin-embedded testis tissues from 20 caucasian male patients: 3 patients with sex-cord stromal tumors (2 Leydig cell tumor and 1 Sertoli cell tumor) (ages from 25 to 31 years) and 17 patients with testicular germ cell tumors (10 seminoma and 7 embryonal carcinoma) (ages from 20 to 35 years) undergoing to therapeutic orchidectomy. Non neoplastic testicular tissues were obtained from 3 caucasian male patients ( ages from 29 to 36 years) showing testes with a granulomatous lesion. The archival cases were provided by the Pathologic Anatomy Unit (Annunziata Hospital, Cosenza, Italy). The ethical committee members of the University of Calabria approved the investigation programme.

### Histopathological analysis

Morphological studies were carried out by Haematoxylin-Eosin staining.

### Chemicals and antibodies

The reagents were purchased from Sigma Aldrich ( Milan, Italy), unless otherwise indicated.

Anti-GPR30 (GPER) primary antibody was rabbit polyclonal LS-A4271(MBL International Corporation, Woburn, MA, USA) which recognizes epitope mapped at the 3^rd ^extracellular domain of human GPR30. Anti- human GATA- 4 was goat polyclonal C-20 (Santa Cruz Biotechnology, Santa Cruz, CA ). Rabbit polyclonal anti β-actin (Santa Cruz Biotechnology, Ca, USA) was also used as loading control. Biotinylated goat-anti-rabbit IgG (Vector Laboratories, INC, Burlingame, CA), biotinylated rabbit- anti -goat (Vector Laboratories, INC, Burlingame, CA), goat anti-rabbit horseradish peroxidase conjugated IgG (Amersham, USA ) were used as secondary antibodies.

### Immunohistochemical analysis

Paraffin embedded sections, 5 μm thick, were mounted on slides precoated with poly-lysine, and then they were deparafinized and dehydrated (7-8 serial sections). Immunohistochemical experiments were performed after heat-mediated antigen retrieval. Hydrogen peroxide (3% in distilled water) was used, for 30 minutes, to inhibit endogenous peroxidase activity while normal goat serum (10% ) was utilised, for 30 minutes, to block the non-specific binding sites. Immunodetection was carried out using anti-GPR30 (GPER) (1:100) primary antibody at 4°C overnight. Then, a biotinylated goat-anti-rabbit IgG was applied (1:600) for 1 hour at RT, followed by the avidin-biotin-horseradish peroxidase complex (ABC/HRP) (Vector, Laboratories, CA, USA). Immunoreactivity was visualized by using the diaminobenzidine chromogen (DAB) (Zymed Laboratories, CA, USA). Testis sections were also counterstained with haematoxylin. The primary antibody was replaced by normal rabbit serum in negative control sections. Absorption controls have utilised primary antibodies preabsorbed with an excess (5 nmol/ml) of the purified blocking peptide (MBL International Corporation, Woburn, MA, USA), at 4°C for 48 hours. Breast cancer tissue was used as positive control.

With the aim to identify the Sertoli cells, sections of control testes were also subjected to the procedure outlined above using anti-human GATA- 4 (1: 50) as primary antibody.

#### Scoring system

The immunostained slides of tumour samples were evaluated by light microscopy using the Allred Score [24], which combines a proportion score and an intensity score. A proportion score was assigned representing the estimated proportion of positively stained tumor cells ( 0 = none; 1 = 1/100; 2 = 1/100 to <1/10; 3 = 1/10 to <1/3; 4 = 1/3 to 2/3; 5 = >2/3). An intensity score was assigned by the average estimated intensity of staining in positive cells (0 = none; 1 = weak; 2 = moderate; 3 = strong). Proportion score and intensity score were added to obtain a total score that ranged from 0 to 8. A minimum of 100 cells were evaluated in each slide. Six to seven serial sections were scored in a blinded manner for each sample. The one-way ANOVA was used to evaluate the differences in the scores between tumor and control samples.

### Protein extraction

Protein extraction from formalin-fixed paraffin-embedded sections was carried out according to Ikeda [25]. Briefly, 50 μm testis sections were deparaffinized in xylene, dehydrated in graded ethanol, immersed in distilled water, and air dried. Then, the selected area was recovered from the glass slides, further it was cut into small pieces and placed in Eppendorf tubes. Two hundred μl of RIPA buffer, pH 7,6 (1 M NaH_2_PO_4_, 10 mM Na_2_HPO_4_, 154 mM NaCl, 1% Triton X-100, 12 mM C_24_H_39_O_4_Na, 0,2% NaN_3_, 0,95 mM NaF, 2 mM PMSF, 50 mg/ml aprotinin, 50 mM leupeptin) containing 0,2% SDS, was added to each tube and the contents were incubated at 100°C for 20 minutes, followed by incubation at 60°C for 2 hours. After incubation, tissue lysates were centrifuged at 15,000 × g for 20 minutes at 4°C and the supernatants were stored at -80°C until biochemical analysis.

### Western blot analysis

Tissue lysates were quantified using Bradford protein assay reagent [26]. Equal amounts of protein (50 μg) were boiled for 5 minutes, separated under denaturing conditions by SDS-PAGE on 10% polyacrylamide Tris-glycine gels and electroblotted to nitrocellulose membrane. Non-specific sites were blocked with 5% non fat dry milk in 0.2% Tween-20 in Tris-buffered saline (TBS-T) for 1 hour at RT and incubated overnight with anti-GPR30 (GPER) (1:500), anti-βactin (1:1000) primary antibodies. The antigen-antibody complexes were then detected by incubation of the membranes for 1 hour at RT with the horseradish peroxidase-conjugated secondary antibodies (1:7000). The bound secondary antibodies were located with the ECL Plus Western blotting detection system (Amersham, USA) according to the manufacturer's instruction. Each membrane was exposed to the film for 2 minutes. Breast cancer tissue were used as positive control. Negative controls were prepared using tissue lysates, where antigens were previously removed by pre-incubation with specific antibodies (1 hour at room temperature) and subsequently immunoprecipitated with protein A/G -agarose.

WB analysis was repeated 3 times for each sample

## Results

### Morphological study

#### Control testes

**Figure 1:**
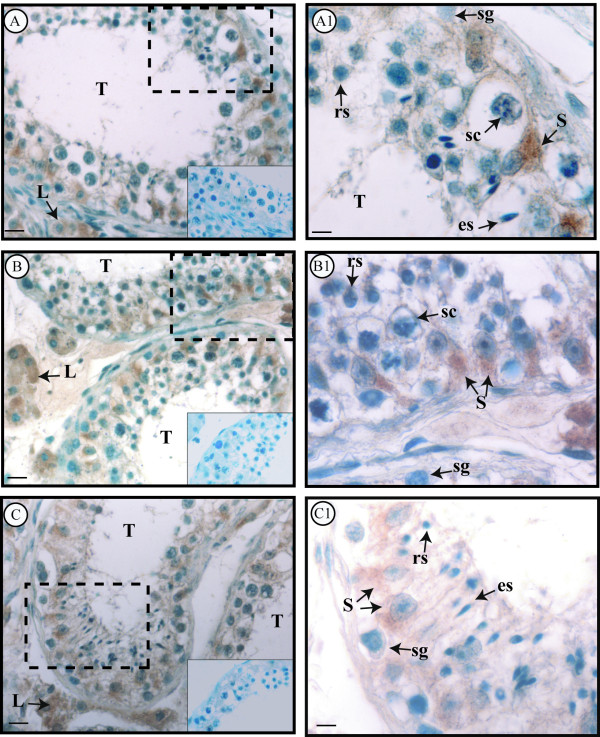
**Immunolocalization of GPER in control testes**. A-C: Positive GPER immunoreactivity in the somatic cell cytoplasm of the three samples. A1-C1: Higher magnifications of testicular areas surrounded by the dashed lines in A-C. **L**, Leydig cell; **T**, seminiferous tubule; **S**, Sertoli cell; **sg**, spermatogonium; **sc**, spermatocyte; **rs**, round spermatid; **es**, elongated spermatid. Inserts: absorption controls. Scale bars = 12.5 μm (A, B, C), 5 μm (A1, B1,C1).

The unaltered regions of non neoplastic testes were used as control samples. They displayed typical seminiferous tubules showing active spermatogenesis. In the basal compartment of seminiferous tubules, Sertoli cells were identified for their typical characteristics: large irregular nuclei with distinct nucleoli and extensive cytoplasmic processes extending from the basement membrane to the lumen of the tubule. Furthermore, Leydig cells were observed in the interstitial tissue (Figure 1).

**Figure 2:**
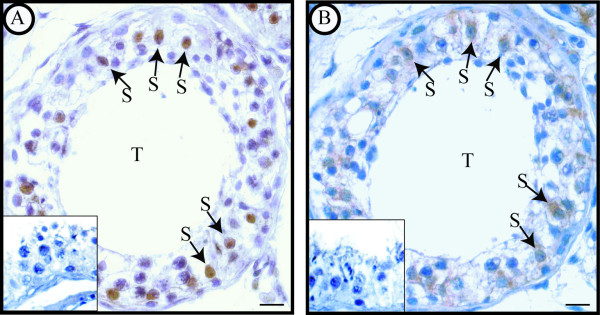
**Immunostaining of GATA-4 and GPER in control testis (serial sections)**. A. Dark GATA-4 nuclear immunoreactivity in Sertoli cells (arrows). B. Dark GPR30 staining in the cytoplasm of Sertoli cells (arrows). Scale bars = 12.5 μm.

In addition to morphological analysis, identification of Sertoli cells was supported by their dark nuclear staining of GATA-4, a Sertoli cell marker [27]. Figure 2 shows a representative comparison between the immunostainings of GATA-4 (2 A) and GPER (2 B) in two serial sections of control testis. The same result was observed in all control samples.

#### Tumor testes

##### Sex cord- stromal tumors

**Figure 3:**
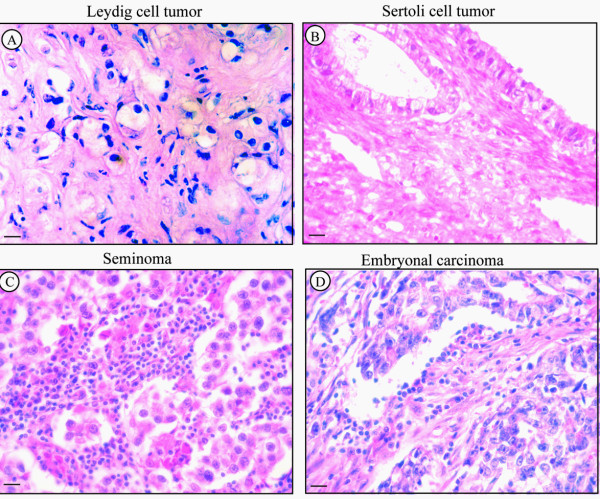
**Haematoxylin -Eosin staining of representative human testicular tumors**. A. Leydig cell tumor. B. Sertoli cell tumor. C. Seminoma. D. Embryonal carcinoma. Scale bars = 12.5 μm.

Leydig cell tumors revealed large, vacuolated Leydig cells arranged in multiple clusters. In the Sertoli cell tumor a pattern of diffuse tubular differentiation was observed, while neoplastic cells showed pale and vacuolated cytoplasms with dysvolumetric and pyknotic nuclei (Figure 3)

#### Testicular germ cell tumors (TGCTs)

Pure seminoma samples showed uniform populations of round/polygonal big neoplastic cells with well-defined borders. These cells were arranged in diffuse sheets separated by thin septae. Extensive leukocyte infiltrations were observed in all samples (Figure 3). Pure embryonal carcinomas revealed nodular areas surrounded by connective tissue and showing large neoplastic cells with ill-defined borders, big nuclei, pale cytoplasms (Figure 3)

### Immunohistochemistry

The specificity of the GPR30 (GPER) antibody (LS-A4271), used in the present study, was assessed in previous works knocking down the protein expression by a shGPR30 (GPER) [19,28-30]. However, similar results were obtained utilizing the rabbit polyclonal anti-GPR30 (GPER) primary antibody (sc-48524) from Santa Cruz (Santa Cruz Biotechnology, Santa Cruz, CA ) [4,31].

#### Control testes

GPER immunoreactivity was detected in the cytoplasm of Leydig cells and Sertoli cells of the control testes while all germ cells were unlabelled (Figure 1: A, A1, B, B1, C, C1).

#### Tumor testes

**Figure 4:**
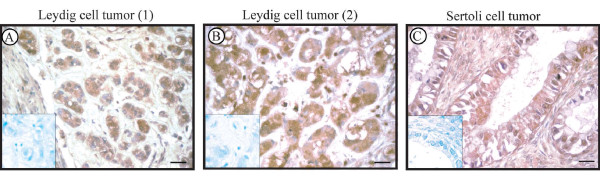
**Immunolocalization of GPER in sex cord- stromal testis tumors.** A-B: Leydig cell tumors, C:Sertoli cell tumor. Inserts: absorption controls. Scale bars = 12.5 μm.

**Figure 5:**
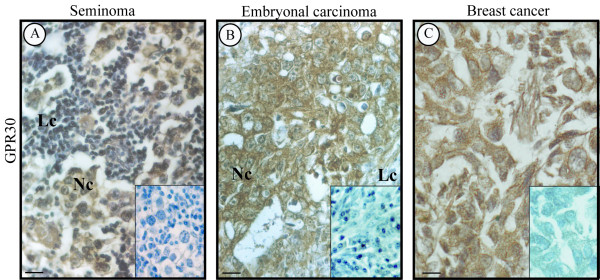
**Immunolocalization of GPER inTGCTs**. A: seminoma. B: embryonal carcinoma. Nc: neoplastic cells. Lc: lymphocytes. C: breast cancer (positive control). Inserts: absorption controls. Scale bars = 12.5 μm.

A positive GPER immunostaining was revealed in neoplastic cell cytoplasm of Leydig and Sertoli cell tumors ( Figure 4: A-B Leydig cell tumors; C Sertoli cell tumor) as well as in all seminoma and embryonal carcinoma samples, while the leukocytes were immunonegative. (Figure 5: A seminoma, B embryonal carcinoma). Table 1 shows the intensity staining scores of GPER in tumor and control samples. As expected, a strong GPER signal was detected in the breast cancer tissue, used as positive control (5 C). Furthermore, negative controls (data not shown) and absorption controls (inserts) were all immunonegative.

**Table 1:** Allred score of GPER in tumor and control testicular cells

Germ cell tumors		Stromal tumors		Control testes	
**Seminoma**		**Leydig cell tumor**		**Germ cells**	
*patient *1	7	*patient *1	7	*control *1	0
*patien*t 2	6	*patien*t 2	6	*contro*l 2	0
*patient *3	7	**Sertoli cell tumor**		*contro*l 3	0
*patient *4	7	*patient *1	7	**Leydig cells**	
*patient *5	7			*control *1	7
*patient *6	5			*control *2	7
*patient *7	7			*contro*l 3	7
*patient *8	6			**Sertoli cells**	
*patient *9	7			*control *1	7
*patient *10	5			*control *2	7
**Embryonal carcinoma**				*control *3	7
*patient *1	6				
*patient *2	6				
*patient *3	5				
*patient *4	5				
*patient *5	5				
*patient *6	6				
*patient *7	6				

Immunostainig score: Total score = Proportion score+Intensity score (range 0-8) TGCT *versus *control = p < 0.001 (one-way Anova test)

### Western blot analysis

**Figure 6:**
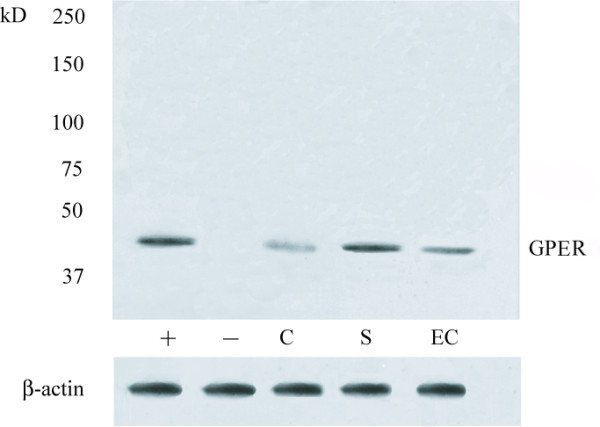
**GPER immunoblots of representative protein extracts from testicular samples**. Positive control *(lane +*), negative control *(lane -*), control testis *(lane C*), seminoma *(lane S *), embryonal carcinoma *(lane EC*). β-actin serves as a loading control. *Numbers *on the left correspond to molecular weight markers.

The immunoblots of testis extracts showed a single band of ~42 kDa in control (Figure 6: *lanes C*) and in testicular germ cell tumor samples (Figure 6: *lanes S, EC *) A band at the same mobility was observed in the positive control (breast cancer) (Figure 6: *lane +*), while the negative control lane was unlabelled (Figure 6: *lane -*). Similar results were obtained from all control and TGCT samples. The quantity of Leydig cell tumor and Sertoli cell tumor samples was not sufficient to perform the protein extraction for Western blot analysis.

## Discussion

It is well known that estrogens regulate growth, differentiation and function of normal human testes through the mediation of estrogen receptor beta isoforms (ERβ1/ERβ2) [1,32]. In addition, previous studies indicated that the classical ERs are also involved in the responses to estrogens in testicular germ cell tumors [33,34] as well as in Leydig cell tumor [35].

The present study identified GPER in non neoplastic and neoplastic cells of human male gonads. Particularly, the "novel" estrogen receptor was localized in the cytoplasm of gonadic cells, according to previous observations showing its presence in the endoplasmic reticulum of different cell types [4,36].

Concerning non neoplastic testes, our investigation evidenced GPER exclusively in somatic cells, i.e. in interstitial Leydig cells and in intratubular Sertoli cells. Particularly, Sertoli cell identification inside seminiferous tubules was supported by the dark nuclear GATA-4 staining; in fact GATA-4 is a transcription factor expressed in Sertoli cells but not in germ cells of adult mammals [27]. On the contrary, in the same samples, GPER immunoreactivity was not observed in germ cells. Therefore, comparing the cellular distribution of GPER between human and rodent testes, it appears that GPER expression is similar in somatic cells but not in germ cells. In fact, GPER (GPR30) has been found in rat Sertoli cells [37] but also in mouse spermatogonia GC-1 cell line [38], in rat pachytene spermatocytes and in rat round spermatids [39,40]. It is interesting to note that a similar species- specific expression has been reported for ERα which has been shown in rat testes[32,41,42] but not in human testes.

Noteworthy, the present study revealed the expression of GPER in testis tumors deriving from both somatic and germ testicular cells. Concerning testicular stromal neoplasms, we identified for the first time, GPER in Leydig cell tumor and Sertoli cell tumor, expanding the limited knowledge of estrogen signaling mechanism in these rare neoplasms. Therefore, the present investigation demonstrated that GPER could mediate estrogen action in both normal and transformed somatic cells of human testes. Conversely, our previous study has evidenced a differential expression pattern of the classical ERs in human normal and neoplastic Leydig cells with the exclusive presence of ERα in tumor cells, which could amplify estrogen signaling and could contribute to tumor growth [35]

Furthermore, the present investigation demonstrated the GPER expression in neoplastic cells of seminoma and embryonal carcinoma. These are testicular germ cell tumors (TGCTs) deriving from abnormal gonocytes which arrest their differentiation and undergo a malignant transformation [43-45]. Our previous paper has demonstrated that ERβ1 and ERβ2 could mediate estrogen action in early and late seminoma and embryonal carcinoma cells [34] as well as in germ cells of control testes. Conversely, the present results revealed GPER in the transformed cells of seminoma and embryonal carcinoma but not in control germ cells. These findings might suggest a possible link between GPER and testis carcinogenesis. In this regard, a very recent study [46] have reported the GPR30(GPER) expression in TGCTs evidencing an increasing expression of the novel estrogen receptor during the tumor development. The authors have suggested GPR30 as a potential therapeutic target.

Interestingly, it has been reported that estrogens can contribute to human germ cell cancer proliferation (JKT-1 seminoma cell line) through a membrane non classical ER [47]. The same authors have also evidenced that the xenoestrogen bisphenol A can promote human seminoma cell proliferation activating PKA and PKG via a membrane G-protein-coupled estrogen receptor [48,49]

Different studies have revealed that GPER and the classical ERs can be co-expressed in some cells, so synergic/antagonist interactions can be expected on the basis of the cellular contexts. In this regard, a recent paper have reported the co-expression of ERβ and (GPR30)GPER in uterine carcinoma together with their significant correlation during tumor progression [50]. In the present and our previous studies [34,35] we evidenced the expression of either the classical ERs or GPER in sex-cord stromal and germ cell testicular tumors, but a possible coordinate regulation or a cross talk between these estrogen receptors will be clarified by future investigations performed on neoplastic samples in early-advanced stages of the disease.

## Conclusions

The present investigation identified the cellular expression of G protein-coupled estrogen receptor (GPER) in non neoplastic and neoplastic human testes. GPER was detected exclusively in Leydig cells and Sertoli cells of non neoplastic testes while it was observed in the transformed cells of Leydig cell tumor, Sertoli cell tumor, seminoma and embryonal carcinoma samples. These findings suggest that GPER could mediate estrogen signaling in both normal and transformed somatic cells of the human testis, but at the same time these results reveal a differential expression of the novel estrogen receptor in normal and neoplastic germ cells.

## Competing interests

The authors declare that they have no competing interest.

## Authors' contributions

VR carried out immunohistochemical expriments and data analysis. FR the author responsible for histoplathological diagnosis. FG carried out Western blot analysis. MM the author responsible for a critical revision of the manuscript. AC the author responsible for conception, design, analysis and interpretation of data as well as of drafting manuscript. All authors read and approved the final manuscript.
